# Primary Urachal Hydatid Cyst in a Child: A Case Report

**Published:** 2019

**Authors:** Tugay TARTAR, Unal BAKAL, Mehmet SARAC, Ibrahim AKDENIZ, Ahmet KAZEZ

**Affiliations:** Department of Pediatric Surgery, School of Medicine, Firat University, 23119, Elazig, Turkey

**Keywords:** Hydatid cyst, Anterior abdominal wall, Urachus, Child, Turkey

## Abstract

The hydatid cyst (HC) is an endemic parasitic disease worldwide. Although the HC can locate in every part of a body, it rarely occurs over the abdominal wall. A 12-year-old female patient was brought to Department of Pediatric Surgery, Firat University School of Medicine, Elazig, Turkey in 2017. She had been suffering from abdominal pain for one week. A lump was determined underneath her skin in the suprapubic region. It was swollen, tense and movable. A cystic mass filling the midline was found in the radiological bladder superior. It was an anechoic cyst causing ondulation on the muscles of the anterior abdominal wall. The sizes of the mass were measured approximately as 9×7 cm (mesentery cyst?). The cystic mass was occurred in the urachal area of the anterior abdominal wall, not in the abdomen. After the cyst was emptied with applying mini median incision below the umbilicus, we saw the germinative membrane inside the cyst. Diagnosis of the HC was confirmed with the pathologic evaluation. For the differential diagnosis of a pure cystic mass, which can locate in every part of a body, diagnosis of the HC should be considered.

## Introduction

*T*he hydatid cyst (HC) is an endemic parasitic disease worldwide. It develops because of the *Echinococcus granulosus* larvae locating in the tissue. The HC is mostly seen in the communities where the livestock industry is widespread (1). It is often found in liver (60%–75%) and lung (15%–25%) (1, 2). However, due to the circulation, it can also be seen in every part of a body such as brain, spleen, kidney, pancreas, musculoskeletal system, uterus, breast, or skin (1–7). Rates of the multiple organ involvement can go up to the percentage of 18% (1, 2, 8).

To our knowledge, there has been no presented case about the urachal HC in the English and Turkish literature. Here we present a rarely seen HC occurring in the urachus.

## Case Presentation

Written informed consent was obtained from parent of the patient who participated in this study.

**Fig. 1:**
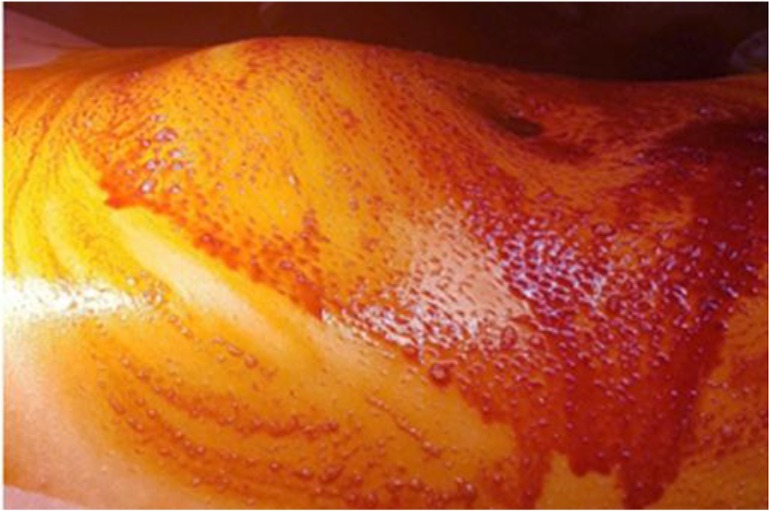
On the abdominal wall, the bump depending on the cyst below the umbilicus

**Fig. 2:**
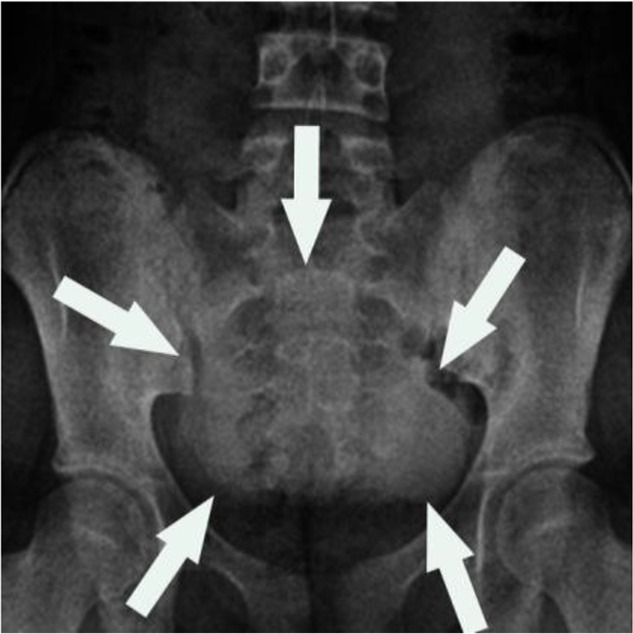
Radioopaque area in the standing direct abdominal radiograph

A 12-year-old female patient was brought to Department of Pediatric Surgery, Firat University School of Medicine, Elazig, Turkey in 2017. She had been suffering from abdominal pain in her suprapubic region for one week. In her physical examination, a lump having smooth surface was determined underneath the skin in the suprapubic region (Fig. 1). It was swollen, tense, partially firm and movable. The diameter of the mass was measured approximately 10 cm. Her hemogram and blood biochemistry were within the normal ranges. There was opacity in the standing direct abdominal radiograph (Fig. 2).

**Fig. 3:**
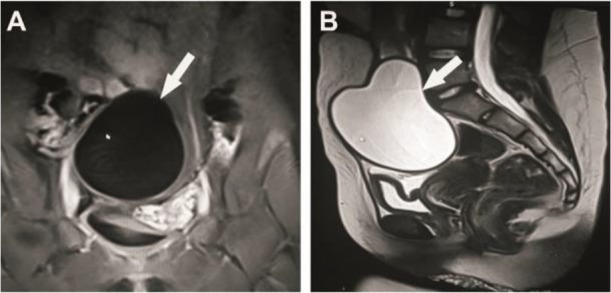
The MR image of the cystic mass (A: T1A, the coronal section pressured by fat, B: T2A, the sagittal section)

Considering the ultrasonography (US), there was anechoic cystic lesion with the sizes of 6.5×9.1 cm in her bladder superior. The thickness of the cyst wall was 2 mm and the vascularisation was not monitored on the wall. The differential diagnosis was mesenteric cyst, lymphocyst, and urachal cyst because of its existence in the midline. In MRI of the lower abdomen, a cystic mass, which completely filled the midline from the promontorium to the anterior abdominal wall, was determined (Fig. 3). It was pure cystic, contrasted, and measured as 9.7×7.8 cm (mesentery cyst?). The mass caused ondulation on the muscles of the anterior abdominal wall. Preoperative intravenous cefotaxime and metronidazole were started. Due to the early diagnosis of mesenteric cyst, laparoscopy was applied at the beginning of the surgery. The cystic mass was not in the intraabdominal, it was seen in the urachal area of the anterior abdominal wall over the periton. Approximately 300 cc of cyst fluid was emptied with an injector from the abdominal wall. This process reduced the tension of the cyst. The cystic mass was not in the abdomen so the laparoscopy was stopped.

**Fig. 4:**
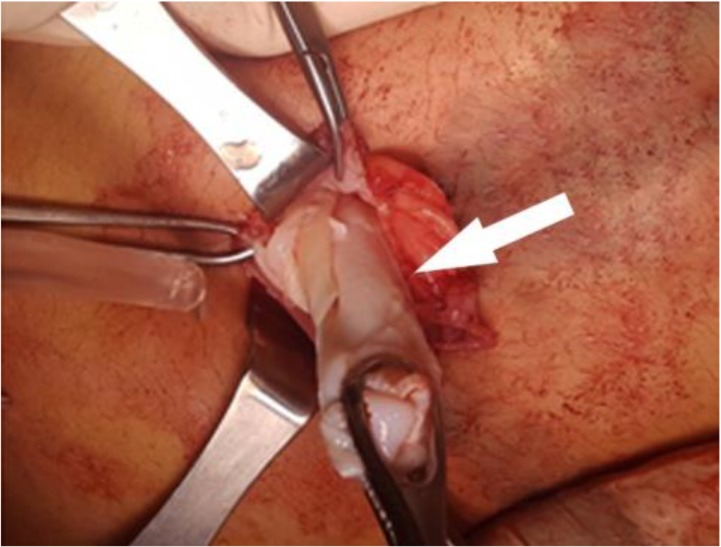
The germinative membrane (shown with the arrow)

We decided to continue with open surgery. Anatomic layers were passed with mini median incision below the umbilicus. A smoothly finite cystic mass, occurred between the rectus muscles, was seen. It was lying until the periton and bladder. The sizes of the mass were approximately measured as 10×10 cm. It was much excised. There was no contact between the mass and bladder. There was germinative membrane inside the cyst (Fig. 4). We washed and cleaned inside of the cyst and its neighbouring tissues with 3% NaCI. Diagnosis of the HC was confirmed with histopathologic evaluation. Five days later after the surgery, the patient was discharged from the hospital with postoperative albendazole treatment.

## Discussion

The hydatid cyst is usually asymptomatic and late diagnosed. Therefore, it can reach large sizes (9). Its symptoms change depending on the complications and the organ where the HC occurs. A physical examination is not enough for the diagnosis. The HC occurring in intraabdominal cause’s abdominal pain and mass; or the cyst located in lung causes coughing, respiratory distress and chest pain (1). Depending on the cyst rupture, anaphylaxis and mortality were also reported (10). In our case, the cyst was asymptomatic until it had large sizes. In addition, because of the lower sociocultural level of the family and applying the hospital lately, the HC reached to very large sizes, macroscopically seen on the abdominal wall. In the physical examination of our case, the cyst was evaluated as a mass with solid component because it was quite tense and partially hard in a narrow region.

Because of its atypical settlement, diagnosis of the HC was not considered. Although the eosinophilia (34%) can be seen in the blood of a patient diagnosed as the HC, this symptom is not specific for the HC (8). In our case, level of the eosinophilia was within the normal range. Reliability of the used serological tests is also not high but they can be meaningful to follow up a patient in terms of relapse (11, 12). The HC was not considered for the early diagnosis in our presented case. Therefore, the indirect hemagglutination test assay was not applied. For the diagnosis, radiological methods such as direct radiography, US, computed tomography (CT) and MRI are used. Compared to the direct radiography and US, the CT gives more prominent information for evaluation of the HC, definition of its complications and differential diagnosis (8, 13). The cyst with thick wall, germinative membrane, daughter cyst and calcifications on the wall are radiological good symptoms for the diagnosis of the HC. However, discrimination of the HC from other simple cysts is difficult when the HC is in its first stage (clear fluid inside anechoic cysts) and the cyst wall is thin. In the direct radiography of our presented case, there was no valuable information expect the opacity occurred by the cyst. We considered the possibilities of mesentery cyst, lenfocystic and urachal cyst as an early diagnosis because the cyst wall was thin (2 mm) and the HC was in its first stage in the US and MRI. However, the HC was not considered as the differential diagnosis.

The main goal of the treatment is removing the fluid and cavity of the cyst and preventing the contamination. Medical treatment (albendazole, mebendazole), Puncture, Aspiration, Injection and Respiration (PAIR), endoscopic and open surgery are the possible applicable methods for the treatment. PAIR is not preferred for children because of sedation, contamination and the risk of anaphylaxis due to this contamination. However, it can be used for the patients who do not respond the medical treatment, have relapsed and are not operated (14–15). The endoscopic operations are not preferred because the lack of data especially for children, contamination, and complications depended on the contamination (15). The endoscopic operations should be preferred because they are minimal invasive methods; they reduce the length of hospital stay and support patient comfort (16). In the presented case, we did not consider the HC so a medical treatment was not begun. Considering the mesentery cyst, we started with the laparoscopic method. After we determined that the cyst did not have contact with the abdomen, open surgery was decided to continue. The laparoscopic methods are minimal invasive methods. Therefore, we offer the usage of the laparoscopic methods for the abdominal HC cases. The laparoscopy can help for the correct determination of pathologies, which leads to the decision of correct surgical method.

## Conclusion

Despite the improvements in the imaging methods, atypically located HC may not be determined clearly. Its asymptomatic course, lack of laboratory findings, and rare body parts where the HC occurs can cause late diagnosis. Especially in the endemic regions, when there is a pure cystic lesion anywhere in a body, the HC should be considered as a differential diagnosis.
